# Histone Deacetylase HDA15 Restrains PHYB-Dependent Seed Germination via Directly Repressing *GA20ox1/2* Gene Expression

**DOI:** 10.3390/cells11233788

**Published:** 2022-11-26

**Authors:** Feng Zheng, Yahan Wang, Dachuan Gu, Xuncheng Liu

**Affiliations:** 1 Key Laboratory of South China Agricultural Plant Molecular Analysis and Genetic Improvement, South China Botanical Garden, Chinese Academy of Sciences, Guangzhou 510650, China; 2 College of Life Sciences, University of Chinese Academy of Sciences, Beijing 100049, China

**Keywords:** histone deacetylation, HDA15, light, PHYB, seed germination, GA biosynthesis

## Abstract

Seed germination is essential for the colonization of the land plants. Light is a major environmental factor affecting seed germination, which is predominantly regulated by photoreceptor phytochrome B (PHYB). PHYB is activated by red light (designated as PHYB-on) whereas it is inactivated by far-red light (referred as PHYB-off). We previously reported that *Arabidopsis* histone deacetylase HDA15 interacts with phytochrome-interacting factor1 (PIF1) to repress seed germination under PHYB-off conditions. Here, we show that HDA15 plays a negative role in regulating seed germination under PHYB-on conditions. Overexpression of *HDA15* in *Arabidopsis* restrains PHYB-dependent seed germination, while gibberellin (GA) relieves the repressive role of HDA15 under PHYB-off conditions. We further show that HDA15 directly binds to *GA20ox1* and *GA20ox2*, two key GA biosynthesis genes and represses their expression by removal of histone H3 and H4 acetylation. Moreover, the levels of *HDA15* transcript and HDA15 protein are up-regulated in the *phyB* mutant. Collectively, our work proposes that HDA15 acts as a negative regulator of PHYB-dependent seed germination by directly repressing *GA20ox1/2* gene expression.

## 1. Introduction

Seed germination is the first step for seed plants to initiate a new life cycle, which is precisely regulated by diverse endogenous factors and exogenous signals. Light is one of the most important environmental cues for plant growth and development, which is perceived by multiple types of photoreceptors, such as phytochromes, cryptochromes, phototropins and UVR8 [1,2]. When deciding whether or not to germinate, plant seeds monitor various environmental factors including light, and translate these conditions into signals transmitted by plant hormones, such as gibberellin (GA), abscisic acid (ABA), brassinosteroid, and ethylene [3,4]. GA and ABA control seed germination in opposite directions, with ABA inhibiting and GA promoting the completion of germination. High levels of GA induce the degradation of DELLA proteins and thereby promote the completion of seed germination [5,6].

Phytochromes are the major photoreceptors that promote germination [7,8]. Phytochromes are synthesized in the cytosol in their inactive form known as Pr. Upon red light (R) absorption, Pr converts to the biologically active Pfr form. *Arabidopsis* genome encodes five phytochromes, PHYA-PHYE [9], among which, PHYB plays a predominant role in the initial phase of seed germination [7,10]. PHYB is activated by red light (designated as PHYB-on) whereas inactivated by far-red light (referred as PHYB-off) in imbibed seeds [11]. Light-activated PHYB promotes seed germination partly by destabilization of phytochrome-interacting factor1 (PIF1), a master repressor of light-regulate seed germination [4,12]. PIF1 directly induces the expression of *DELLA* genes, *RGA* and *GAI*, or indirectly increases the transcription of GA catabolic and ABA anabolic-related genes via downstream factor somnus (SOM) [4,11].

Emerging evidences revealed that light-regulated plant development involves dynamic chromatin reprogramming. Reversible histone acetylation and deacetylation, which catalyzed by histone acetyltransferases (HATs) and histone deacetylases (HDACs), play an important role in modulating chromatin structure and transcription activity [13,14,15]. Histone deacetylase15 (HDA15), a RPD3/HDA1-type histone deacetylase, associates with multiple light-regulated transcription factors such as PIF3, HY5 and NF-YCs in photomorphogenesis [16,17,18]. Furthermore, HDA15 was identified as a negative regulator of seed germination under PHYB-off conditions. HDA15 interacts with PIF1 and forms a transcription module to repress the expression of seed germination-related genes [19]. However, the biological role of HDA15 under PHYB-on conditions remains unclear.

In present work, we found that HDA15 plays a negative role when PHYB is activated during seed germination, and GA relieves the repression role of HDA15. HDA15 directly represses the expression of GA biosynthesis genes, *GA20ox1* and *GA20ox2*, by decreasing the levels of histone H3 and H4 acetylation. Moreover, the transcription of *HDA15* and protein accumulation of HDA15 are upregulated in *phyB* mutant. Our work uncovers a novel role of HDA15 in PHYB-dependent seed germination.

## 2. Materials and Methods

### 2.1. Plant Materials

All Arabidopsis seeds used in this study are in Col-0 background. The *hda15-1* and *phyB-9* mutants were obtained from TAIR center. The *HDA15-OE1* and *OE2* were as described in previous report [19]. The seeds used for light-dependent germination, seeds were harvested in the same batch of plants grown at 22 °C under long days (16 h WL/8 h dark). Following harvesting, seeds were dried in an incubator at 22 °C for about 1 month prior to germination assays.

### 2.2. PHYB-Dependent Seed Germination Assays

The PHYB-dependent seed germination assays were performed as described previously [20]. Briefly, seeds were surface-sterilized and plated on half-strength Murashige-Skoog (Sigma-Aldrich, MO, USA) agar plates containing 0.3% sucrose and 1% phytoagar (pH 5.7). The plates were placed in an illuminated incubator with white light (100 μmol m^−2^ s^−1^) at 22 °C. After 1 h white light exposure, seeds were irradiated with far-red light (3.8 μmol m^−2^ s^−1^) for 5 min following irradiation with red light (13.1 μmol m^−2^ s^−1^) for 5 min (referred as FR/R or PHYB-on). The seeds were then kept in the dark either for 12 h for the gene expression and ChIP analyses, or kept in the dark for 5 days to score the germination rates. At least 50 seeds were used for each experimental point, and three biological replicates were used for statistical analysis.

### 2.3. RNA Isolation and qRT-PCR Analysis

After FR/R treatment, the seeds were incubated in the dark at 22 °C for 12 h. About 0.1 g seeds for each sample were used for the assay. Total RNA was extracted with TRIZOL Reagent (Invitrogen, Shanghai, China) according to the manufacture’s protocol. After DNAse I treatment, the first strand cDNA was synthesized using 2 μg total RNA according to the manufacturer’s instruction of TransScript One-Step gDNA Removal and cDNA Synthesis Super Mix Kit (TransGen, Beijing, China). 100 ng synthesized cDNA was used as a template to perform real-time RT-PCR analysis. PCR reactions were performed in the total volume of 20 µL, with 0.5 µL for each primer (final concentration 100 nM) and 10 µL for SYBR^®^ Green PCR Supermix (Bio-Rad Laboratories, CA, USA) on a ABI7500 Real-Time PCR System (Applied Biosystems, MA, USA). The PCR program included an initial denaturation step at 94 °C for 3 min, followed by 40 cycles of 5 s at 94 °C and 1 min at 60 °C. Each sample was quantified at least in triplicates and normalized using *UBQ10* as an internal control. All PCR reactions were normalized using Ct value corresponding to the reference gene *UBQ10*. The relative expression levels of target genes were calculated with formula 2^−ddCt^. Values represented the average of three biological replicates. The gene-specific primer pairs for quantitative Real-Time PCR were listed in Supplemental Table S1.

### 2.4. ChIP Assays

ChIP assays were performed as previous described with minor modifications [19]. The seeds were treated with cross-link buffer (1% formaldehyde) under a vacuum for 1 h. 0.2–0.3 g seeds for each sample were used. The chromatin was extracted and sheared to an average length of 500 bp by sonication, and agarose gel electrophoresis was performed to ensure the proper size of the sheared chromatin. Then the chromatin was immunoprecipitated with anti-histone H3ac (Catalog no 06-599, Millipore, MA, USA), anti-histone H4ac (Catalog no 06-866, Millipore, MA, USA) or anti-HDA15 antibodies [16], respectively. The cross-linking was then reversed and the amount of each immunoprecipitated DNA fragment was determined by quantitative PCR using gene specific primers (Supplementary Table S1).

### 2.5. Western Blot Assays

After FR/R treatment, the seeds were kept in the dark for 12 h. Total proteins were extracted with an extraction buffer (50 mM Tris-HCl at pH 7.4, 150 mM NaCl, 2 mM MgCl_2_, 1 mM dithiothreitol, 20% glycerol, and 1% CA-630) containing protease inhibitor cocktail (Roche). After the proteins were separated in 10% SDS-PAGE gel, the endogenous levels of histone H3 and HDA15 were detected by anti-histone H3 (Catalog no 05-499, Millipore, MA, USA) and anti-HDA15 antibodies [16], respectively.

### 2.6. Statistical Analysis

Statistical differences were assessed by a Student’s *t*-test using SPSS 13.0. Values of *p* < 0.05 were considered statistically significant (compared with wild-type). Data are presented as mean ± SD.

## 3. Results

### 3.1. HDA15 Restrains PHYB-Dependent Seed Germination While GA Relieves Its Repressive Role

**Figure 1:**
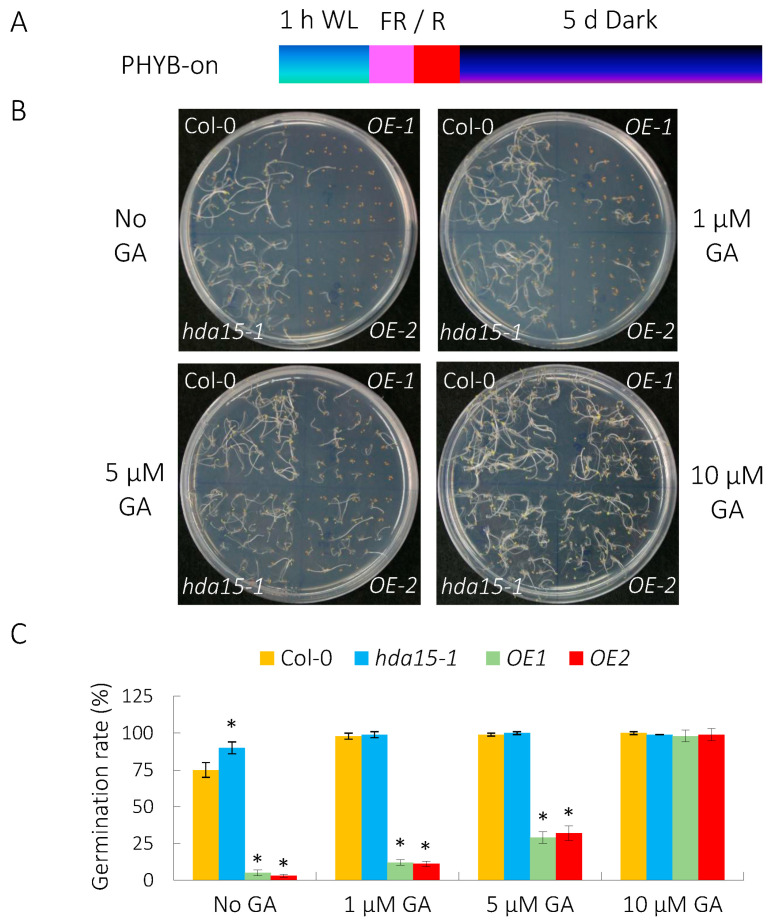
GA treatment relieves the repressive role of HDA15 in PHYB-dependent seed germination. (**A**) Germination protocols of PHYB-dependent (PHYB-on) assays. FR/R, 5 min of far-red light (3.8 µmol m^−2^ s^−1^) and then 5 min of red light (13.1 µmol m^−2^ s^−1^). The imbibed seeds were kept under white light (WL) for 1 h and were subsequently irradiated with FR/R and kept in the dark for 5 d. (**B**) Germination patterns of *hda15* mutant and *HDA15* overexpression lines (*OE1* and *OE2*) under PHYB-on conditions. (**C**) Germination rates of *hda15* mutant and *HDA15* overexpression lines under PHYB-on conditions. Different concentrations (0, 1, 5 and 10 µM) of GA (GA_3_) were added in the plates before treatment, respectively. At least 50 seeds were used for each sample and performed in triplicate. Values are shown as means ± SD. (Student’s *t*-test, * *p* < 0.05, n = 3).

We have previously showed that HDA15 represses seed germination under PHYB-off conditions [19]. To investigate the role of HDA15 in PHYB-on conditions, we analyzed the germination rates of *HDA15* mutant *hda15-1* [16], and *HDA15* overexpression lines *HDA15-OE1* and *OE2* [19] (Supplemental Figure S1). For PHYB-dependent germination assays, seeds were illuminated with far-red light followed with red light (referred as FR/R or PHYB-on), and then kept in the dark for 5 days (Figure 1A). *hda15-1* displayed a significant elevated germination rate (90%) compared with wild-type (75%), whereas 5% and 3% of *HDA15-OE1* and *OE2* seeds germinated respectively (Figure 1B,C), revealing HDA15 plays a negative role in seed germination under PHYB-on conditions.

Since GA is key plant hormone promoting seed germination, we further tested the effect of GA treatment on the germination of *HDA15-OE* seeds under PHYB-on conditions. Different concentrations of GA_3_ (0, 1, 5, 10 µM) were added into the plates prior to light treatment. GA treatments gradually increased the germination rates of *HDA15* overexpression lines, furthermore, almost all of the seeds germinated upon 10 µM GA treatment (Figure 1B,C). These data suggest that HDA15 may repress PHYB-mediated promotion of seed germination via GA biosynthesis pathway.

### 3.2. HDA15 Regulates the Expression of GA and ABA Metablism-Related Genes

**Figure 2:**
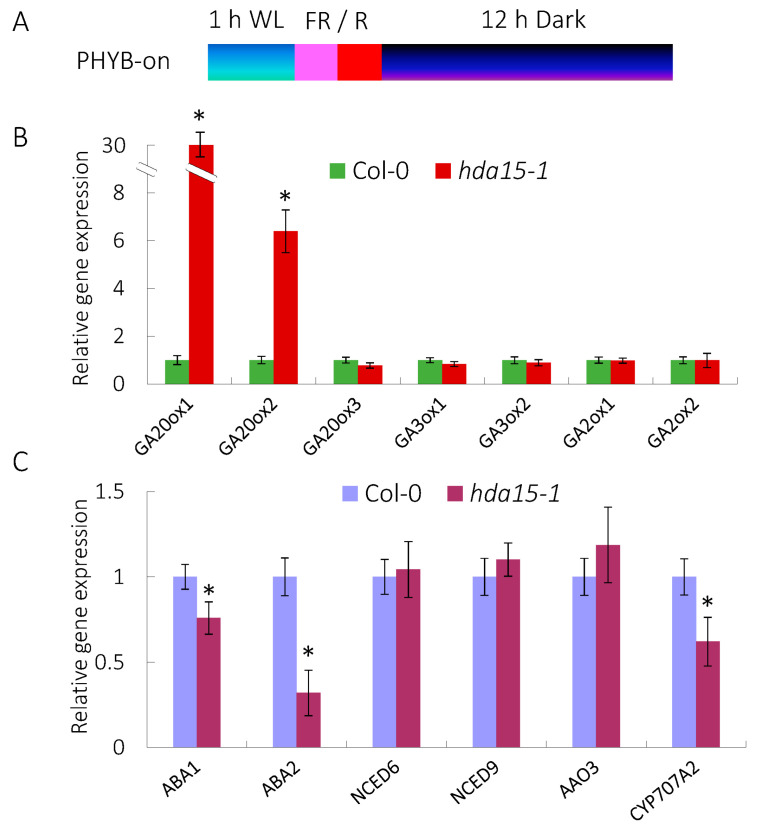
HDA15 regulates the expression of GA and ABA metabolism-related genes under PHYB-on conditions. (**A**) Diagram of PHYB-on conditions. FR/R, 5 min of far-red light (3.8 µmol m^−2^ s^−1^) and then 5 min of red light (13.1 µmol m^−2^ s^−1^). After FR/R irradiation, the seeds were kept in the dark for 12 h. (**B**) Gene expression patterns of GA metabolism-related genes in Col-0 and *hda15-1*. (**C**) Gene expression patterns of ABA metabolism-related genes in Col-0 and *hda15-1* seeds. *UBQ10* was used as an internal control. Values are shown as means ± SD (Student’s *t*-test, * *p* < 0.05, n = 3).

To further explore the role of HDA15 in PHYB-mediated promotion of seed germination, we examined the expression levels of GA and ABA metabolism-related genes in *hda15* mutant. After FR/R treatment, seeds were kept in the dark for 12 h and then harvested for RNA isolation and quantitative RT-PCR (qRT-PCR) assays (Figure 2A). The transcripts of two GA biosynthesis genes, *GA20ox1* and *GA20ox2* were significantly up-regulated, by 26.3 and 6.4 folds compared to those in the wild-type, respectively, whereas the expression levels of other GA biosynthesis genes *GA20ox3*, and *GA3ox1*, as well as the GA catabolism genes, *GA2ox1* and *GA2ox2* remained unchanged in the *hda15* mutant (Figure 2B). Furthermore, the transcripts of two ABA biosynthesis genes, *ABA1* and *ABA2* were significantly down-regulated in the *hda15* mutant, with 0.76 and 0.32 times the level of those in the wild-type, respectively (Figure 2C). In addition, the ABA catabolism gene *CYP707A2* was also down-regulated in the *hda15* seeds compared with the wild-type (Figure 2C). Together, the above data indicate that HDA15 regulates the gene expression of GA and ABA metabolism genes under PHYB-on conditions.

### 3.3. HDA15 Directly Targets GA20ox1 and GA20ox2 in Imbibed Seeds

**Figure 3:**
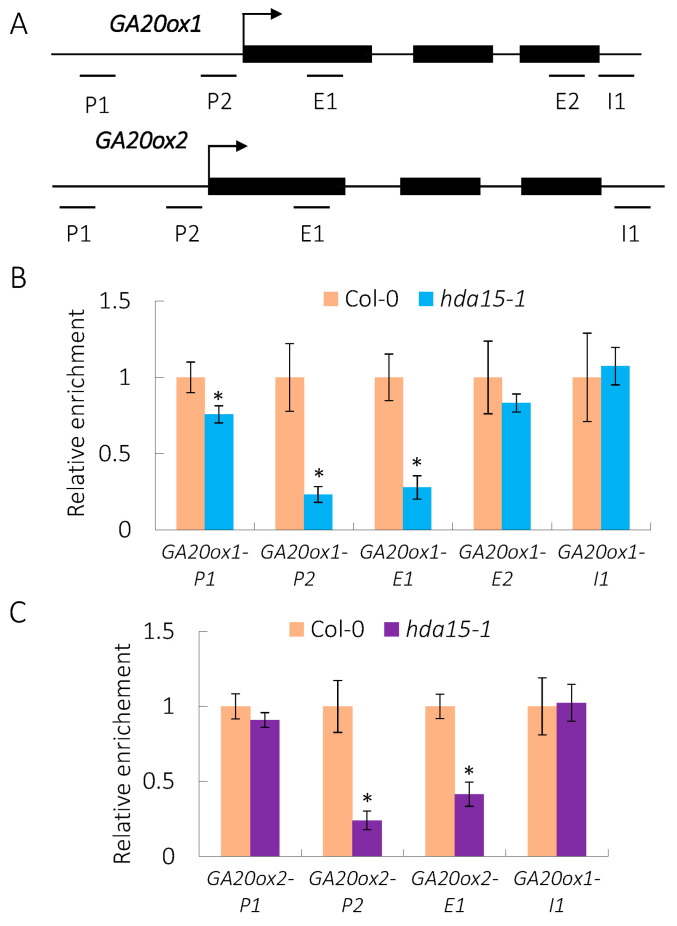
HDA15 directly targets the promoter and exon regions of *GA20ox1* and *GA20ox2* in imbibed Arabidopsis seeds under PHYB-on conditions. (**A**) Schematic structures of *GA20ox1* and *GA20ox2* genes. (**B**,**C**) ChIP-qPCR analysis of relative HDA15 enrichment to the promoter and gene body regions of *GA20ox1* and *GA20ox2* in Col-0 and *hda15-1* seeds under PHYB activation conditions. An anti-HDA15 antibody was used for immunoprecipitation. The amounts of DNA after ChIP were quantified and normalized to *ACTIN2*. Values are shown as means ± SD (Student’s *t*-test, * *p* < 0.05, n = 3).

Histone deacetylases usually act as transcriptional repressors in eukaryotes [13]. Up-regulated expression of *GA20ox1* and *GA20ox2* in *hda15-1* under PHYB-on conditions promoted us to investigate whether these two genes are direct targets of HDA15 in vivo. The enrichment of HDA15 in the promoter, exon and intron regions of *GA20ox1* and *GA20ox2* was detected by chromatin immunoprecipitation (ChIP) assays (Figure 3A). An anti-HDA15 antibody [16] was used for immunoprecipitation. The levels of HDA15 proteins on the P1 and P2 promoter regions and E1 exon region of *GA20ox1* in *hda15* were 0.75, 0.23 and 0.28 times those in the wild-type, respectively (Figure 3B). Similarly, the levels of HDA15 protein on the P2 promoter region and E1 exon region of *GA20ox2* in *hda15* were 0.24 and 0.41 times those in the wild-type respectively (Figure 3C). These data reveal that *GA20ox1* and *GA20ox2* are direct targets of HDA15 in imbibed seed under PHYB-on conditions.

### 3.4. HDA15 Decreases the Levels of Histone Acetylation of GA20ox1 and GA20ox2

**Figure 4:**
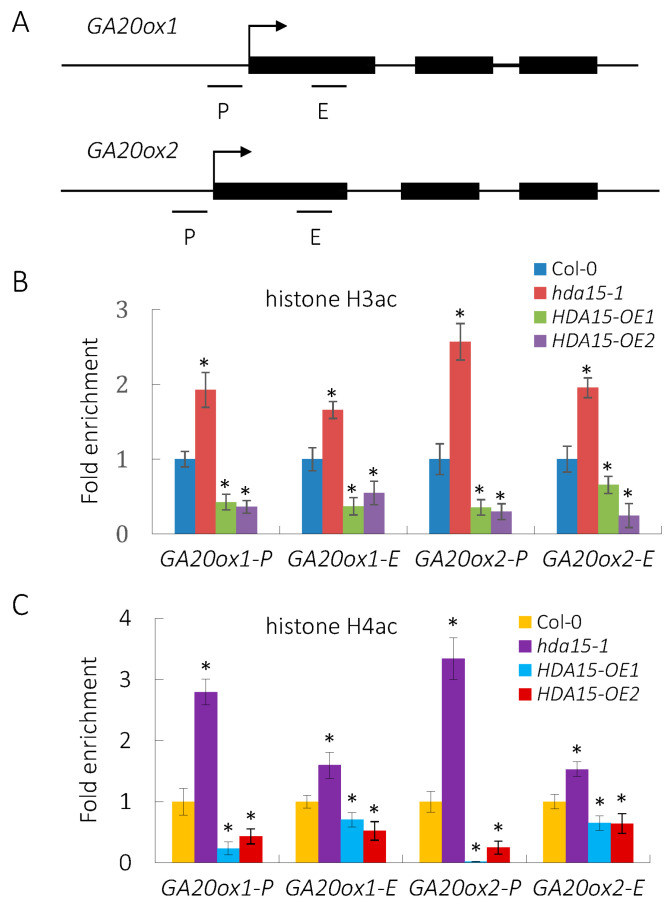
HDA15 decreases the histone acetylation level of *GA20ox1* and *GA20ox2* in Arabidopsis seeds under PHYB-on conditions. (**A**) Schematic structures of *GA20ox1* and *GA20ox2* genes. (**B**) ChIP-qPCR analysis of the relative histone H3ac levels in imbibed Col-0, *hda15-1*, *HDA15-OE1* and *HDA15-OE2* seeds. (**C**) ChIP-qPCR analysis of the relative histone H4ac levels in imbibed Col-0, *hda15-1*, *HDA15-OE1* and *HDA15-OE2* seeds. The amounts of DNA after ChIP were quantified and normalized to *ACTIN2*. Values are shown as means ± SD (Student’s *t*-test, * *p* < 0.05, n = 3).

HDA15 has been reported to possess histone deacetylase activity both in vitro and in vivo [18]. Therefore, we examined whether HDA15 acts to decease the histone acetylation levels of its targets, *GA20ox1* and *GA20ox2* in imbibed seeds. The regions related to proximal promoters and first exons of these genes were detected (Figure 4A). ChIP assays showed that both the histone H3 and H4 acetylation levels at the proximal promoter and exon regions of *GA20ox1* and *GA20ox2* were significantly up-regulated in the *hda15* mutant; in contrast, the histone H3 and H4 acetylation levels at these regions were significantly down-regulated in *HDA15-OE1* and *HDA15-OE2* seeds compared with wild-type (Figure 4B,C). Taking these data together, our findings reveal that HDA15 represses the expression of *GA20ox1* and *GA20ox2* by removal of their histone acetylation.

### 3.5. PHYB Affects the Gene Expression of HDA15 and Protein Level of HDA15

**Figure 5:**
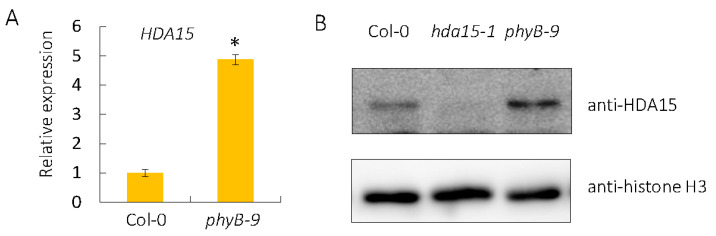
PHYB represses the gene expression and protein accumulation of HDA15 in imbibed seeds under PHYB-on conditions. (**A**) qRT-PCR analysis of the expression levels of *HDA15* in Col-0 and *phyB-9* mutant under PHYB-on conditions. *UBQ10* was used as an internal control. Values are shown as means ± SD (Student’s *t*-test, * *p* < 0.05, n = 3). (**B**) Western-blot analysis of HDA15 protein levels in Col-0 and *phyB-9* mutant under PHYB-on conditions. Histone H3 was used as an internal control.

The negative role of HDA15 in PHYB-dependent seed germination prompted us to detect whether PHYB affects the gene expression of *HDA15* and protein accumulation of HDA15 in imbibed seeds. First, the transcription levels of *HDA15* in Col-0 and *PHYB* mutant *phyB-9* [21] were analyzed. The level of *HDA15* transcripts in *phyB-9* mutant was 4.87 times the level in the wild-type (Figure 5A). Consistently, the protein level of HDA15 was also up-regulated in *phyB-9* mutant (Figure 5B). Collectively, these data suggested that PHYB may promote seed germination partly by decreasing the transcription level of *HDA15*.

## 4. Discussion

### 4.1. HDA15 Plays Dual Roles in PHYB-Dependent Seed Germination

R/FR photoreversible effect on seed germination was first reported in *Lacuca sativa* [8]. Later, it was found that the phytochromes play a major role in the photoregulation of seed germination. In Arabidopsis, PHYB controls seed germination while PHYA plays only a supplementary role in the absence of PHYB [7]. The PHYB-dependent seed germination assays were designed based on the principle that R irradiation induces the inactive Pr form of PHYB to assume the active Pfr form whereas FR exposure reverses this process in imbibed seeds. Photo-activated PHYB promotes seed germination partly by interacting and thereby destabilizing the master germination repressor PIF1 [20,22].

In the previous study, we demonstrated that HDA15 interacts with PIF1 to co-repress a large subset of genes related to plant hormones and cellular processes via histone deacetylation under PHYB-off conditions. Upon R exposure, the activated form of PHYB translocates into the nucleus and induces rapid degradation of PIF1 and dissociation of HDA15 from these targets [19]. Previous work clearly uncovered the role of HDA15 in seed germination when PHYB is inactivated in imbibed seeds. In present work, we focused on the role of HDA15 when PHYB is activated. *HDA15* overexpression restrains seed germination, whereas GA relieves its repressive effect. Consistently, we showed that HDA15 represses the expression of the GA biosynthesis genes *GA20ox1*/*2*, indicating that HDA15 inhibits PHYB-dependent seed germination via repressing GA biosynthesis pathway. Collectively, these findings reveal that HDA15 regulates seed germination under both PHYB-on and PHYB-off conditions by repressing the expression of distinct target genes.

### 4.2. HDA15 May Interact with Site-Specific GA-Signal Related Transcription Factors to Repress GA20ox1/2 Expression

The final steps of GA biosynthesis are catalyzed by GA20 oxidases (GA20ox) and GA3-oxidases (GA3ox), which convert inactive GA precursors into bioactive GAs [23]. In the present study, we showed that HDA15 represses *GA20ox1/2* expression by decreasing the levels of histone H3 and H4 acetylation. Unlike other transcription regulators, HDAC proteins lack a DNA binding motif, and they usually function as transcriptional co-repressor through interacting with other proteins [13]. This suggests that HDA15 may be recruited to *GA20ox1/2* by some sequence-specific transcription factors.

Recent works reported that GAI-associated factor1 (GAF1), a DELLAs interacting transcription factor, directly binds to the consensus sequence TTTTGTCG in the promoters of *GA20ox1* and *GA20ox2* [24,25,26]. Interestingly, in addition to DELLAs, GAF1 also associates with the TOPLESS (TPL)-related protein complex [24], an important transcriptional corepressor, which has been shown to be associated with HDA6 and HDA19 in regulating multiple development processes [27,28,29]. Therefore, HDA15 may associate with GAF1 transcription factor as well as TPL corepressor to repress the expression of *GA20ox1/2*. Further research could be performed to detect the interaction of HDA15 with GAF1 in the regulation of *GA20ox1/2* expression.

In previous work, we found that HDA15 targets another GA biosynthesis genes *GA3ox1* and *GA3ox2* under PHYB-off conditions [19]. Since HDA15 has no DNA binding activity, it associates with the downstream genes dependent on different transcription factors [16,17,18]. The specific regulation mode of HDA15 on different GA biosynthesis genes might be dependent on the protein levels of the specific transcription factors which recruit HDA15 to different targets. Further identification of the HDA15-interacting proteins under different light conditions may help to elucidate the specific role of HDA15 in light-regulated seed germination.

In yeast and mammals, HDACs are identified to be components of multi-protein co-repressor complexes, such as SIN3, NuRD, CoREST and NCoR/SMRT [30]. A recent work co-purified HDA15 with the SIN3 homologue SNL2 (SIN3-LIKE2) [31], a scaffold protein playing a role in control of seed germination [32], which suggested that HDA15 may associate with SNL2-containing protein complex in repressing *GA20ox1/2* expression. In summary, HDA15 may associate with GA signal-related transcription factors and co-repressor complexes in repressing *GA20ox1/2* expression in the regulation of PHYB-dependent seed germination.

Photo-activated PHYB promotes seed germination via both up-regulating the expression of GA biosynthesis genes and down-regulating the expression of ABA biosynthesis genes [33,34]. In the present work, we showed that the transcription levels of *GA20ox1* and *GA20ox2* are up-regulated while the other GA metabolism genes including *GA3ox1*, *GA3ox2*, *GA2ox1* and *GA2ox2* are not significantly altered in the *hda15* mutant, which suggests that HDA15 may specifically regulate some of the GA biosynthesis genes in imbibed seeds. Furthermore, we showed that the expression of the ABA metabolism genes *ABA1*, *ABA2* and *CYP707A2* are down-regulated in *hda15* mutant. HDA15 was reported to possess histone deacetylase activity and usually act as transcriptional repressor of the downstream genes [16,17,18], which indicated that HDA15 may indirectly regulate the expression of these ABA metabolism-related genes. Collectively, these data reveal that HDA15 inhibits PHYB-dependent seed germination via directly or indirectly regulating GA and ABA metabolism pathways.

### 4.3. PHYB May Promote Seed Germination Partly through Decreasing the Transcription of HDA15

Among the five phytochromes, PHYB plays a major role in light-regulated seed germination processes [10]. PHYB activates seed germination partly by interacting with and destabilizing PIF1, the master repressor of light-regulated seed germination [22]. The MYB-type transcription factor REVEILLE1 (RVE1) is another master regulator of PHYB-mediated seed germination, which directly inhibits the expression of *GA3ox2* and promotes DELLA protein RGL2 accumulation [33,34]. A recent study showed that ERF-type transcription factors ERF55/ERF58 play a negative role in light-regulated seed germination. Light-activated PHYA and PHYB repress the expression of *ERF55/ERF58*; furthermore, PHYA and PHYB interact with ERF55/ERF58 and inhibit their binding activity upon the downstream targets, leading to promoted seed germination [35]. These findings suggest that PHYB promotes seed germination through regulating multiple downstream light-responsive factors.

In the present work, gene expression and protein level analyses revealed that PHYB may repress the transcription of *HDA15* under the PHYB-on condition. Decrease of HDA15 protein level under the PHYB-on condition could lead to elevated histone acetylation and expression levels of *GA20ox1/2,* which may ultimately induce seed germination. Further work is need to investigate how HDA15 is regulated by phytochromes under different light conditions.

## Supplementary Materials

The following supporting information can be downloaded at: https://www.mdpi.com/article/10.3390/cells11233788/s1, Figure S1: Quantitative RT-PCR analysis of the levels of *HDA15* transcripts in *HDA15* transgenic plants; Figure S2: Gene expression analysis of GA and ABA biosynthesis genes in imbibed seeds of *HDA15* overexpression lines under PHYB-on conditions; Figure S3: Western-blot analysis of HDA15 protein levels in imbibed Col-0 and *phyB-9* seeds under PHYB-on conditions; Table S1: List of primers used in this study.
