# Supplementary material for: Histone Deacetylase HDA15 Restrains PHYB-Dependent Seed Germination via Directly Repressing GA20ox1/2 Gene Expression
Source: Cells. 2022 Nov 26;11(23):3788. doi: 10.3390/cells11233788 (PMC9735612; doi:10.3390/cells11233788)
Supplement: Supplementary file 1 [file cells-11-03788-s001.zip › Supplemental Figures.pptx]

## Slide 1
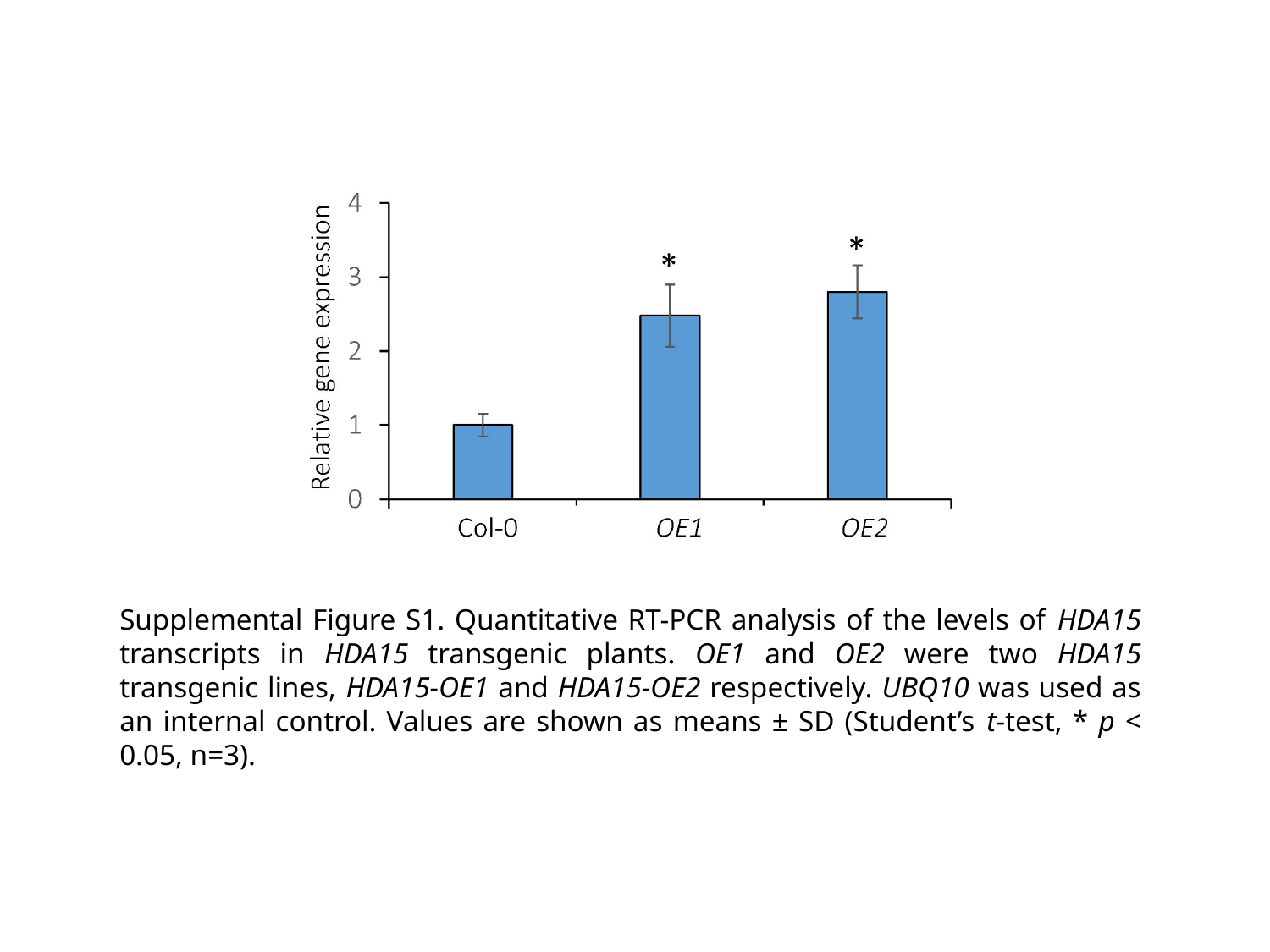

Supplemental Figure S1. Quantitative RT-PCR analysis of the levels of HDA15 transcripts in HDA15 transgenic plants. OE1 and OE2 were two HDA15 transgenic lines, HDA15-OE1 and HDA15-OE2 respectively. UBQ10 was used as an internal control. Values are shown as means ± SD (Student’s t-test, * p < 0.05, n=3).

## Slide 2
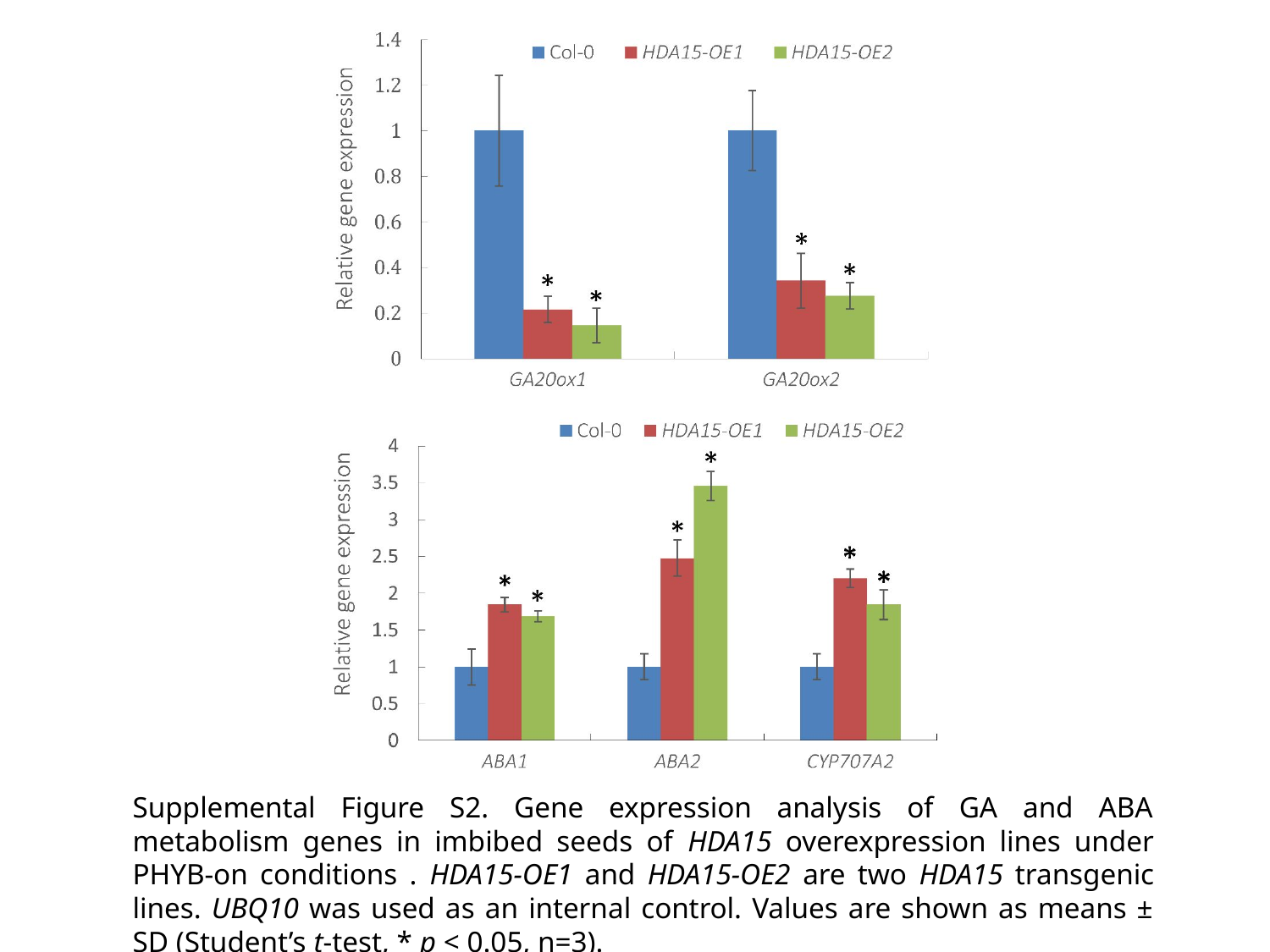

Supplemental Figure S2. Gene expression analysis of GA and ABA metabolism genes in imbibed seeds of HDA15 overexpression lines under PHYB-on conditions . HDA15-OE1 and HDA15-OE2 are two HDA15 transgenic lines. UBQ10 was used as an internal control. Values are shown as means ± SD (Student’s t-test, * p < 0.05, n=3).

## Slide 3
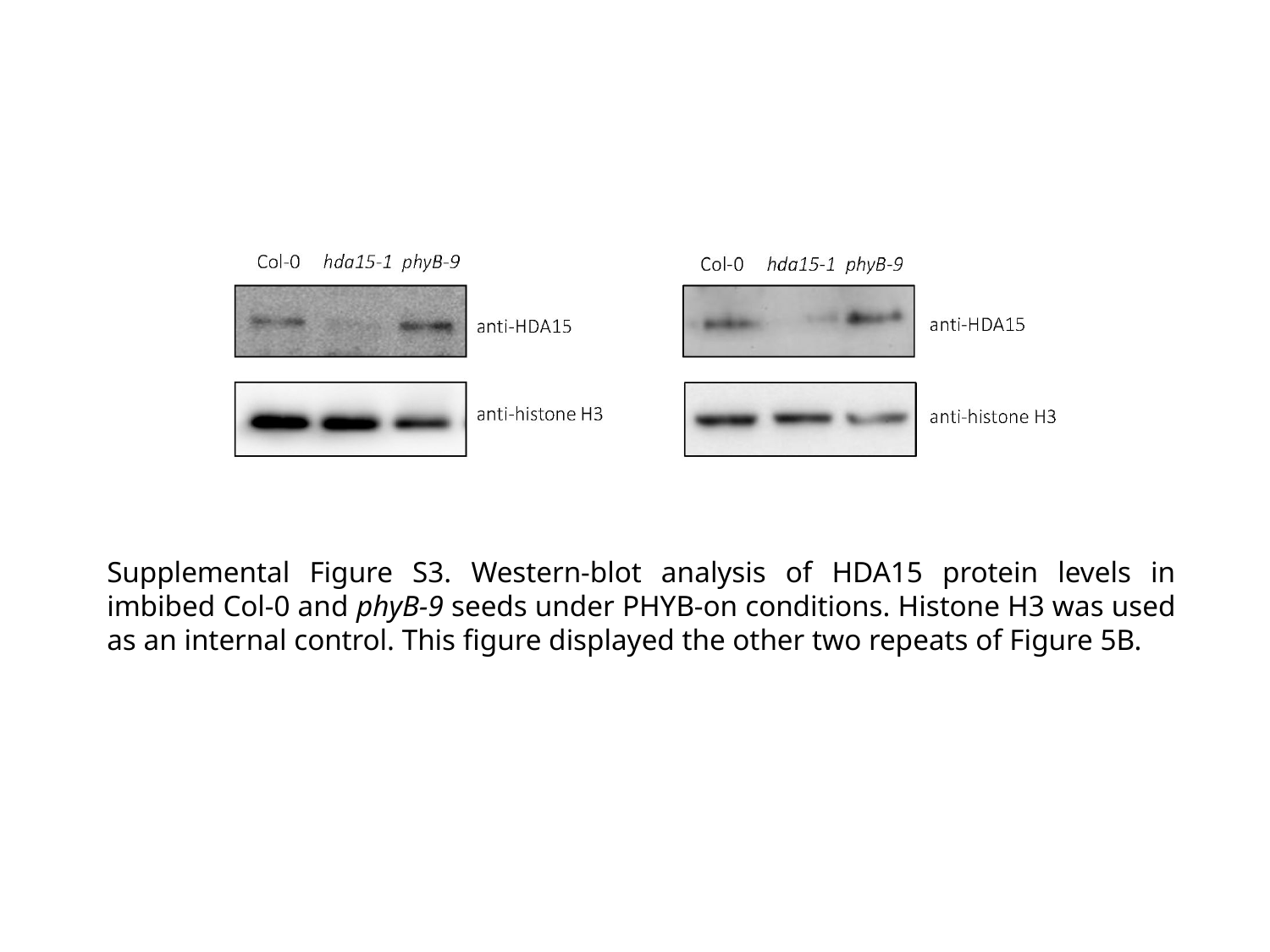

Supplemental Figure S3. Western-blot analysis of HDA15 protein levels in imbibed Col-0 and phyB-9 seeds under PHYB-on conditions. Histone H3 was used as an internal control. This figure displayed the other two repeats of Figure 5B.
